# *Anomaloglossus meansi* sp. n., a new Pantepui species of the *Anomaloglossus beebei* group (Anura, Aromobatidae)

**DOI:** 10.3897/zookeys.759.24742

**Published:** 2018-05-22

**Authors:** Philippe J.R. Kok, Michaël P.J. Nicolaï, Amy Lathrop, Ross D. MacCulloch

**Affiliations:** 1 Amphibian Evolution Lab, Department of Biology, Vrije Universiteit Brussel, Pleinlaan 2, B-1050 Brussels, Belgium; 2 Department of Biology, Vrije Universiteit Brussel, Pleinlaan 2, B-1050 Brussels, Belgium; 3 Centre for Biodiversity and Conservation Biology, Royal Ontario Museum, 100 Queen’s Park, Toronto, Ontario M5S 2C6, Canada

**Keywords:** Aromobatidae, diversity, Guiana Shield, Guyana, Pakaraima Mountains

## Abstract

Recent extinctions and drastic population declines have been documented in the Guiana Shield endemic frog genus *Anomaloglossus*, hence the importance to resolve its alpha-taxonomy. Based on molecular phylogenies, the literature has long reported the occurrence of an undescribed species in the Pakaraima Mountains of Guyana in the Pantepui region. We here describe this new taxon and demonstrate that in addition to divergence at the molecular level the new species differs from congeners by a unique combination of morphological characters, notably a small size (maximum SVL in males 18.86 mm, maximum SVL in females 21.26 mm), Finger I = Finger II when fingers adpressed, Finger III swollen in breeding males, fringes on fingers absent, toes basally webbed but lacking fringes, in life presence of a thin dorsolateral stripe from tip of snout to tip of urostyle, and a black throat in preserved males (immaculate cream in females). Virtually nothing is known about the ecology of the new species. We suggest the new species to be considered as Data Deficient according to IUCN standards.

## Introduction

In their influential work about bird diversification in the Venezuelan highlands, Mayr and Phelps (1967) coined the term “Pantepui” to describe the high-elevation life zones of the Guiana Shield highlands in north-eastern South America. Pantepui is best known for its numerous isolated vertical-sided sandstone table-top mountains, the iconic Lost World’s tepuis, and huge tepuian massifs, last erosional remnants of a vast ancient plateau (see Kok 2013 for details). The number of phylogenetic lineages restricted to Pantepui is remarkable given the relatively reduced size of that bioregion. Pantepui seems to act as a reservoir of endemism at the species level, but also at the genus level and, to a lesser extent, at the family level (see Kok 2013 for summary). Various biogeographical hypotheses have been proposed to explain the origin and drivers of diversification of tepui-summit species/populations (Mayr and Phelps 1967, see Kok 2013 for a summary). Recent phylogeographic studies based on non-flying vertebrates (e.g. Kok et al. 2012, 2017, 2018a, b, Leite et al. 2015, Lehmberg et al. 2018) suggested a complex historical biogeography involving the synergy of long distance dispersals, vicariance and habitat shifts.

**Figure 1.**
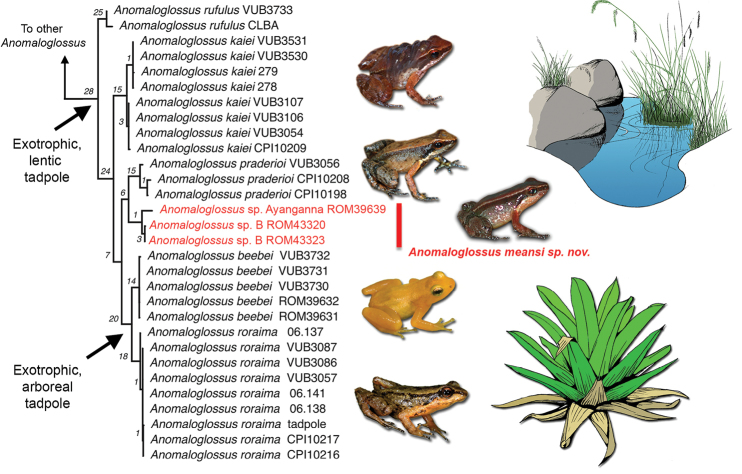
Optimal relationships of the *Anomaloglossus beebei* group (modified from Grant et al. 2017). Numbers at nodes are Goodman-Bremer values. The new species is highlighted in red. *Anomaloglossus* photos are by PJRK, except *A. meansi*, which is by D. Bruce Means. Drawings by Kim Roelants.

Vertical isolation makes tepui ecosystems particularly sensitive to global warming (see Rull and Vegas-Vilarrúbia 2006, Nogué et al. 2009). Because of their remoteness and the difficulties to access most tepuis and tepuian massifs, sampling in the area has been historically low, hindering the pressing need to evaluate the taxonomic status and accurate distribution of Pantepui endemic species. The situation is particularly critical in some groups in which recent extinctions or drastic population declines have been documented, such as in the Guiana Shield endemic frog genus *Anomaloglossus* (e.g. Fouquet et al. 2015, 2018). The genus currently comprises 28 species (Grant et al. 2017, Fouquet et al. 2018), and likely originated in the Pantepui region (area sensu Kok 2013), where several endemics with restricted distributions are reported; more widespread species are found in the lowlands of the eastern Guiana Shield (Vacher et al. 2017). The *beebei* species group (sensu Grant et al. 2017) is restricted to the eastern Pantepui region of Venezuela and Guyana and currently contains six species, one of them still undescribed (Figure 1). That unnamed species has previously been reported in the literature as *Anomaloglossus* sp. Ayanganna (Grant et al. 2006, 2017), Anomaloglossus cf. praderioi (Kok 2010) and as *Anomaloglossus* sp. B (Kok et al. 2012) and was recovered sister to *A. praderioi* (La Marca, 1997) by Grant et al. (2006, 2017) and Kok et al. (2012). The new taxon was first collected in October 2000 by AL and RDM during an expedition to Mount Ayanganna in Guyana, then found on the Wokomung Massif, Guyana, in July 2003 by D. Bruce Means and in October 2004 by AL and RDM. There is no additional report of the species since then. Although its status as an undescribed species has never been disputed, no formal description has yet been proposed. It is our aim to resolve the issue and describe this new species based on the eleven collected specimens currently available.

## Materials and methods

### Nomenclature

Taxonomy and terminology follow Grant et al. (2006, 2017). The description format is adapted from the most recent species (re)descriptions in the genus (e.g., Kok et al. 2006, Myers and Donnelly 2008, Kok 2010, Kok et al. 2010, Fouquet et al. 2015, 2018).

### Fieldwork and deposition of specimens

**Figure 2.**
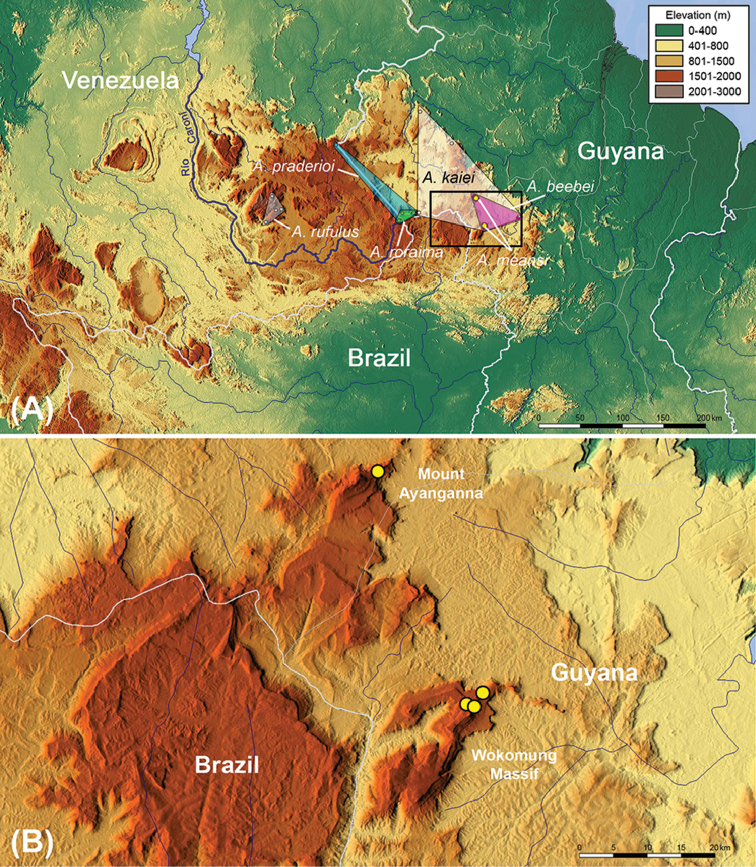
Occurrence map of the *Anomaloglossus* species belonging to the *beebei* group (coloured convex polygons); distribution of *A. meansi* sp. n. is depicted by yellow dots. **A** map of the Eastern Pantepui District; black rectangle is enlarged in **B**. **B** localities of occurrence of *Anomaloglossus meansi* sp. n. based on museum specimens.

Collecting activities took place on Mount Ayanganna and the Wokomung Massif, west-central Guyana (Figure 2). These two mountains, located in the southern Pakaraima range, are the easternmost high tepuis in the Guiana Shield. Ayanganna and Wokomung are 37 km apart, and some anuran species occur on both mountains (e.g. in the genus *Stefania*, MacCulloch et al. 2006), although the degree of species overlap is not yet fully known.

Specimens were collected by hand and euthanized by immersion in a solution of MS 222 (ROM specimens) or by immersion in 20% isopropanol (CPI specimen). Tissue (a piece of liver or thigh muscle) was removed from most specimens immediately after euthanasia and preserved in 95–100% ethanol for later molecular analyses. Whole individuals were fixed in 10% formalin and later transferred to 70% ethanol for permanent storage. Type specimens have been deposited in the collections of the Royal Ontario Museum, Canada (ROM) and the Coastal Plains Institute and Land Conservancy, USA (CPI); tissue samples were deposited in the Amphibian Evolution Lab, Biology Department, Vrije Universiteit Brussel (VUB) and ROM. Coordinates and elevations were acquired using Global Positioning System units and referenced to map datum WGS84.

### Morphology

All morphometric data were taken from the preserved specimens by the same person (MPJN), to the nearest 0.01 mm, under a Leica stereo dissecting microscope using an electronic digital caliper. Colour pattern in life was taken from field notes and colour photographs. Sex and maturity were determined by the presence/absence of vocal slit(s) and by dissection. Comparisons of external character states are based both on original descriptions and examination of museum specimens (see Appendix for comparative material examined). Abbreviations for measurements are as follows:

**SVL** snout-vent length

**HW** head width, at level of angle of jaws

**HL** head length, from angle of jaw to tip of snout

**IOD** inter orbital distance

**EN** eye to naris distance, from anterior edge of eye to centre of naris

**SL** snout length, from anterior edge of eye to tip of snout

**TSL** tip of snout length, from centre of naris to tip of snout

**IND** internarial distance, the distance between the centres of nares

**EL** eye horizontal length

**TYM** tympanum horizontal length

**HAND I–IV** relative lengths of fingers, from the proximal edge of the palmar tubercle to the tip of each finger

**WFD** width of disc on Finger III

**FAL** forearm length, from elbow joint to proximal edge of metacarpal tubercle

**THL** thigh length, from vent opening to flexed knee

**TIL** tibia length, from knee to heel

**TAL** tarsus length, from heel to proximal edge of outer metatarsal tubercle

**FL** foot length, from proximal edge of inner metatarsal tubercle to tip of Toe IV

**WTD** width of disc on Toe IV

## Taxonomy

### Anomaloglossus meansi sp. n.

Taxon classificationAnimaliaAnuraAromobatidae

http://zoobank.org/84C73332-67F9-4412-8140-CF70F1FB419C

**Figure 3.**
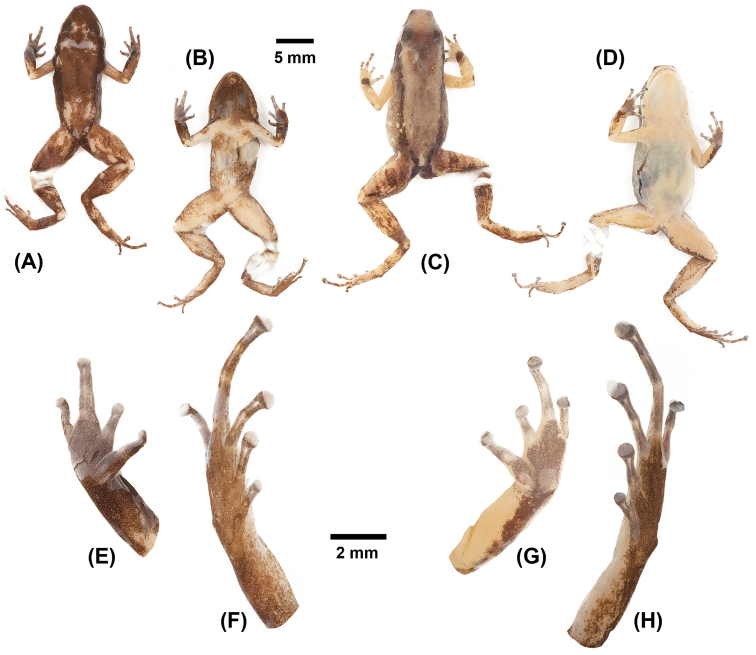
*Anomaloglossus meansi* sp. n. in preservative. **A** male holotype ROM 43896, dorsal view **B** male holotype ROM 43896, ventral view **C** female paratype ROM 43323, dorsal view **D** female paratype ROM 43323, ventral view **E** male holotype ROM 43896, ventral view of right hand **F** male holotype ROM 43896, ventral view of right foot **G** female paratype ROM 43323, ventral view of left hand **H** female paratype ROM 43323, ventral view of left foot.

**Figure 4.**
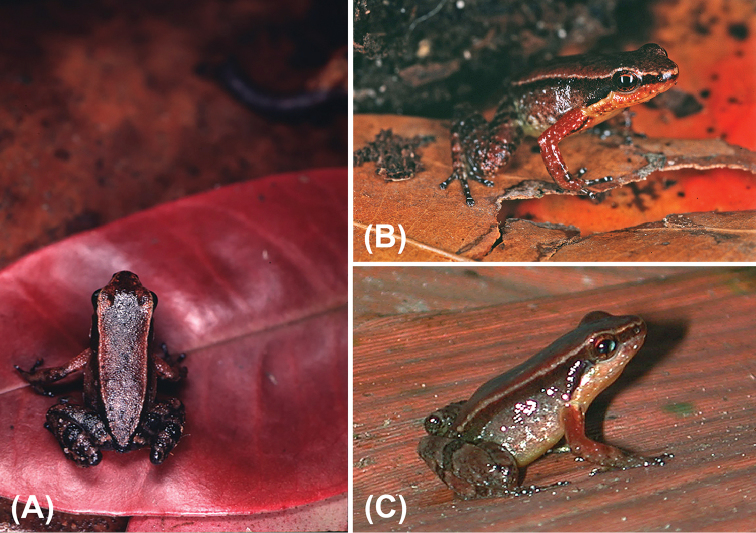
*Anomaloglossus meansi* sp. n. in life. **A** female paratype ROM 43332, dorsal view **B** female paratype ROM 43329, dorsolateral view **C** male paratype CPI 11000, dorsolateral view. Photographs (**A, B**) by AL; photograph (**C**) courtesy D. Bruce Means.

Figures 3 , 4 ; Table 1

Anomaloglossus sp. Ayanganna Grant et al. 2006: 120–121, 2017: S66.
Anomaloglossus cf. praderioi Kok 2010: 66.
Anomaloglossus sp. B Kok et al. 2012: supplementary information.

#### Holotype.

ROM 43896, adult male from the vicinity of Camp 2 on the Wokomung Massif, Potaro-Siparuni District, Guyana (05°06.5833'N; 059°49.2667'W), 1234 m elevation, collected by A. Lathrop and R. James on 30 October 2004.

#### Paratypes

(n = 10). An adult male (ROM 39639) from the northeast plateau of Mount Ayanganna, Cuyuni-Mazaruni District, Guyana (05°24.1'N; 059°57.4'W), 1490 m elevation, collected by R. D. MacCulloch, A. Lathrop and C. Cox on 26 October 2000; four adult females (ROM 43320, ROM 43329, ROM 43331, ROM 43332) from the vicinity of Camp 2 on the Wokomung Massif, Potaro-Siparuni District, Guyana (05°06.5833'N; 059°49.2667'W), 1234 m elevation, collected by A. Lathrop, R. D. MacCulloch and S. Khan between 26–31 October 2004; one adult female (ROM 43323) from the vicinity of Camp 3 on the Wokomung Massif, Potaro-Siparuni District, Guyana (05°05.65'N; 059°50.5833'W), 1411 m elevation, collected by A. Lathrop, R. D. MacCulloch and S. Khan on 3 November 2004; one juvenile (ROM 43322) from the vicinity of Camp 2 on the Wokomung Massif, Potaro-Siparuni District, Guyana (05°06.5833'N; 059°49.2667'W), 1234 m elevation, collected by C. Alban on 26 October 2004; two juveniles (ROM 43324, ROM 43325) from the vicinity of Camp 2 on the Wokomung Massif, Potaro-Siparuni District, Guyana (05°06.5833'N; 059°49.2667'W), 1234 m elevation, collected by A. Lathrop, R. D. MacCulloch and S. Khan between 28–31 October 2004; and one adult male (CPI11000) from Falls Camp on the Wokomung Massif, Potaro-Siparuni District, Guyana (05°05.4333'N; 059°50.2833'W), ca. 1371 m elevation, collected by D. Bruce Means on 24 July 2003.

#### Diagnosis.

The following characteristics pertain to preserved specimens unless otherwise noted. A medium-sized *Anomaloglossus* differing from other species in the genus by the following combination of characters: (1) mean SVL in males 18.53 mm (18.15–18.86 mm, n = 3), mean SVL in females 19.15 mm (17.66–21.26, n = 5); (2) skin on dorsum shagreened, venter smooth; (3) tympanic annulus visible anteroventrally; (4) Fingers I and II subequal in length, FI = FII when fingers adpressed; (5) tip of Finger IV not surpassing the base of the distal subarticular tubercle on Finger III when fingers adpressed; (6) distal subarticular tubercle on Finger III and IV present; (7) Finger III swollen in males (conspicuous pre- and postaxial swelling in breeding males); (8) fringes on fingers absent; (9) toes basally webbed, fringes on toes absent; (10) tarsal keel well defined, slightly tubercle-like and weakly curved at proximal end; (11) black arm gland absent, glandular supracarpal pad present in both sexes (larger and more glandular in males); (12) cloacal tubercles absent; (13) pale paracloacal mark present; (14) in life, thin dorsolateral stripe present, from tip of snout to tip of urostyle (not visible, or only barely distinguishable in preservative); (15) ventrolateral stripe absent, but presence of irregular white blotches on the lower flank; (16) oblique lateral stripe absent; (17) sexual dichromatism in throat colour pattern: throat heavily pigmented with melanophores in males (dark brown to black in life), immaculate cream in females (yellowish-orange in life); (18) sexual dichromatism in ventral colour pattern: belly pigmented with melanophores in males, immaculate cream in females; (19) in life, iris metallic reddish bronze with fine dark brown reticulation; (20) large intestine extensively pigmented; (21) testes cream, unpigmented; (22) mature oocytes partly pigmented; (23) median lingual process small, longer than wide, tapered; (24) maxillary teeth present, small.

#### Comparisons.

*Anomaloglossus meansi* sp. n. can mainly be distinguished from the four described species belonging to the *degranvillei* group [sensu Vacher et al. 2017 and Fouquet et al. 2018, i.e. *A. blanci* Fouquet, Vacher, Courtois, Villette, Reizine, Gaucher, Jairam, Ouboter & Kok, 2018, *A. degranvillei* (Lescure, 1975), *A. dewynteri* Fouquet, Vacher, Courtois, Villette, Reizine, Gaucher, Jairam, Ouboter & Kok, 2018 and *A. surinamensis* Ouboter & Jairam, 2012; characters in parentheses] by having FI = FII when fingers adpressed (FI > FII), the tympanic annulus anteroventrally conspicuous (inconspicuous), and a conspicuous thin dorsolateral stripe from tip of snout to tip of urostyle (absent or inconspicuous).

*Anomaloglossus meansi* sp. n. can mainly be distinguished from the four described species belonging to the *stepheni* group [sensu Vacher et al. 2017, i.e. *A. apiau* Fouquet, Souza, Nunes, Kok, Curcio, Carvalho, Grant & Rodrigues, 2015, *A. baeobatrachus* (Boistel & de Massary, 1999), *A. leopardus* Ouboter & Jairam, 2012 and *A. stepheni* (Martins, 1989); characters in parentheses] in lacking an oblique lateral stripe (present, even if short and discontinuous in *A. apiau*), and in having a conspicuous thin dorsolateral stripe from tip of snout to tip of urostyle (absent).

*Anomaloglossus meansi* sp. n. can mainly be distinguished from the three described species belonging to the *megacephalus* group [sensu Grant et al. 2017, i.e. *A. megacephalus* Kok, MacCulloch, Lathrop, Willaert & Bossuyt, 2010, *A. verbeeksnyderorum* Barrio-Amorós, Santos & Jovanovic, 2010, *A. wothuja* (Barrio-Amorós, Fuentes-Ramos & Rivas-Fuenmayor, 2004); characters in parentheses] in having only basal toe webbing (moderate to extensive), in lacking an oblique lateral stripe (present, even if short and/or discontinuous), and in having a conspicuous thin dorsolateral stripe from tip of snout to tip of urostyle (absent).

Compared to the other five species belonging to the *beebei* group [sensu Grant et al. 2017, i.e. *A. beebei* (Noble, 1923), *A. kaiei* (Kok, Sambhu, Roopsind, Lenglet & Bourne, 2006), *A. praderioi*, *A. roraima* (La Marca, 1997) and *A. rufulus* (Gorzula, 1990)], *A. meansi* sp. n. can easily be distinguished from *A. beebei* by its larger size in males (maximum SVL 18.86 mm in *A. meansi* [n = 3,] versus maximum SVL 16.80 mm [n=18] in *A. beebei*), smooth ventral skin (granular in *A. beebei*), basal toe webbing (moderate in *A. beebei*), and in having a conspicuous thin dorsolateral stripe from tip of snout to tip of urostyle (absent or originating from the posterior corner of eye); from *A. kaiei* in having a conspicuous thin dorsolateral stripe from tip of snout to tip of urostyle (originating from the posterior corner of eye in *A. kaiei*) and a black throat in preserved males (greyish, never black in *A. kaiei*); from *A. roraima* by its larger size in females (maximum SVL 21.26 mm in *A. meansi* [n = 3,] versus maximum SVL 19.30 mm [n = 18] in *A. roraima*), smooth ventral skin (granular in *A. roraima*), and in having a conspicuous thin dorsolateral stripe from tip of snout to tip of urostyle (when present originating from the anterior or posterior corner of eye in *A. roraima*); from *A. rufulus* in having a conspicuous thin dorsolateral stripe from tip of snout to tip of urostyle (absent in *A. rufulus*) and the posterior part of belly unmarked (heavily marbled in *A. rufulus*). *Anomaloglossus meansi* sp. n. is most similar to *A. praderioi* with which it shares a conspicuous thin dorsolateral stripe from tip of snout to tip of urostyle but is immediately distinguished by its smaller size in males (maximum SVL 18.86 mm in *A. meansi* [n = 3,] versus maximum SVL 22.40 mm [n = 11] in *A. praderioi*), the absence of fringes on toes (extensive in *A. praderioi*), Finger III with pre- and postaxial swelling in breeding males (preaxial swelling only in *A. praderioi*), less toe webbing (compare Figure 3 with figure 2 in Kok 2010), and the lack of black spots on chest and lower flanks in males (present in *A. praderioi*).

Compared to the remainder 12 *Anomaloglossus* species not yet assigned to any group [*A. ayarzaguenai* (La Marca, 1997), *A. breweri* (Barrio-Amorós, 2006), *A. guanayensis* (La Marca, 1997), *A. moffetti* Barrio-Amorós & Brewer-Carías, 2008, *A. murisipanensis* (La Marca, 1997), *A. parimae* (La Marca, 1997), *A. parkerae* (Meinhardt & Parmelee, 1996), *A. shrevei* (Rivero, 1961), *A. tamacuarensis* (Myers & Donnelly, 1997), *A. tepequem* Fouquet, Souza, Nunes, Kok, Curcio, Carvalho, Grant & Rodrigues, 2015, *A. tepuyensis* (La Marca, 1997) and *A. triunfo* (Barrio-Amorós, Fuentes-Ramos & Rivas-Fuenmayor, 2004); characters in parentheses], *A. meansi* sp. n. mainly differs in having only basal toe webbing (moderate to extensive), and in having a conspicuous thin dorsolateral stripe from tip of snout to tip of urostyle (absent).

#### Description of the holotype.

Adult male (ROM 43896; Figure 3), 18.58 mm SVL, in suboptimal state of preservation (extensive ventral incisions, dorsal skin locally damaged); body robust; head as wide as long, HL 99.7% of HW, HW 32% of SVL; dorsal skin shagreened; ventral skin smooth; snout moderately long, SL 47% of HL, 128% of EL, round in dorsal view, protruding in lateral view, extending past lower jaw; nares located close to tip of snout, directed posterolaterally, visible from front, barely visible in dorsal and ventral views, EN 28% of HL, 77% of ED, EN 60% of SL, TSL 49% of SL; posterior rim of naris bordered posteriorly by an inconspicuous crescent-shaped ridge; IND 40% of HW; canthus rostralis rounded; loreal region concave; IOD 104% of EL, longer than upper eyelid; postrictal tubercles low and inconspicuous; tympanic membrane inconspicuous, round, concealed posterodorsally by a diffuse supratympanic swelling; tympanic annulus visible anteroventrally, TYM 52 % of EL; choanae small, circular, located anterolaterally. Maxillary teeth present, small. Tongue longer than wide, free posteriorly, with rounded margin, small median lingual process longer than wide, tapered. Vocal slits bilateral, large, extending from edge of tongue to angle of jaw.

Forelimb swollen, robust, 94% of FAL. Ulnar fold absent, metacarpal ridge absent; swollen, glandular supracarpal pad present, heavily pigmented with melanophores; hand moderate in size, 24% of SVL, 75% of HW; relative length of fingers III>II=I=IV; pre- and postaxial swelling on third finger (i.e. Finger III swollen); fingers without fringes; tip of Finger IV not reaching distal subarticular tubercle on Finger III when fingers adpressed; finger discs expanded, wider than long, about 1.4 times width of digit; width of disc on Finger III 0.52 mm; palmar tubercle large, egg shaped, 0.72 mm (larger than Finger III disc), thenar tubercle smaller, elliptical; one or two round to ovoid subarticular tubercles (one each on Fingers I and II, two each on Fingers III and IV, with distal tubercle on Finger IV less conspicuous).

Hind limb robust, moderately long, with heel of adpressed leg reaching posterior corner of eye; skin granular with no cloacal tubercles discernible (but this could be an artefact of preservation); TL 46% of SVL, heels not in contact when hind limbs are flexed at right angle to sagittal plane of body; FL 38% of SVL; relative length of adpressed toes IV>III>V>II>I; Toe I very short, its tip barely reaching the base of subarticular tubercle of Toe II when adpressed; toe discs larger than width of toes; disc on Toe I only slightly larger than width of digit; width of disc on Toe IV 0.67 mm; toes basally webbed, lateral fringes absent; one to three round to ovoid subarticular tubercles (one each on Toes I and II, two each on Toes III and V, and three on Toe IV, with distal tubercle on Toe IV the smallest and least conspicuous). Inner metatarsal tubercle protuberant elliptical, 0.47 mm in length, outer metatarsal tubercle round, protuberant, pigmented, 0.35 mm in diameter. No medial metatarsal tubercle discernible. Tarsal keel slightly tubercle-like and weakly curved at proximal end, extending distally to preaxial edge of Toe I. Metatarsal fold not visible.

#### Colour of holotype in life.

Dorsal ground colour chestnut brown with a short black middorsal line between shoulders. A black line from snout tip through eye, extending dorsolaterally to groin. A narrow pale brown dorsolateral stripe above this line, blending into the chestnut dorsal ground colour. Upper surface of limbs light brown proximally, becoming dark brown distally. Flanks reddish brown with yellow spots on lower flanks. Venter pale brown with dark brown mottling, throat very dark brown to black. Underside of limbs orange-red, changing to dark reddish brown on distal forearms.

#### Colour of holotype in preservative.

After more than 13 years in preservative, dorsal ground colour became dark chestnut brown with a short middorsal black longitudinal line in the scapular region. No other dorsal marking present. Dorsal surface of arms varies from light brown proximally to dark brown, purplish-black towards the granular supracarpal pads. Dorsal surface of legs light brown with darker brown markings. Flanks dark brown to purplish-black with pale spots on lower flanks. Narrow pale brown dorsolateral stripes indistinguishable from dorsal ground colour, although the black dorsolateral stripe remains visible. Throat black, heavily pigmented with melanophores; belly cream, pigmented with melanophores (less densely distributed than on throat). Pale paracloacal marks are visible. Palms dark brown, soles medium brown (Figure 3).

#### Measurements of holotype

**(in mm).** SVL = 18.58; HL = 5.91; HW = 5.93; IOD = 2.26; EN = 1.68; SL = 2.77; TSL = 1.35; EL = 2.16; TYM = 1.12; IND = 2.39; HAND I = 3.06; HAND II = 3.2; HAND III = 4.44; HAND IV = 3.09; WFD = 0.52; FAL = 3.81; THL = 7.80; TIL = 8.61; TAL = 4.48; FL = 7.08; WTD = 0.67.

#### Sexual dimorphism and variation within the type series.

Males are usually smaller than females, 18.15–18.86 mm SVL (n = 3) versus 17.66–21.26 mm SVL (n = 5) in females, with Finger III distinctly swollen in breeding males (Figure 3). Supracarpal pads are less extended and less glandular in females and juveniles than in males. Colouration is sexually dichromatic; throat heavily pigmented black in males (immaculate yellowish-orange in females), and belly yellowish-orange pigmented with melanophores in males (immaculate yellowish-orange in females) (Figure 3). Venter immaculate in juveniles, although small pigmented areas on throat may occur (presumably in juvenile males).

Morphometric variation is summarized in Table 1, illustrations of a male and a female paratype in life are in Figure 4. Snout in dorsal and ventral views varies from round to truncate (the latter more particularly in females, see Figure 3).

**Table 1.** Morphometric measurements (in mm) of the type series of *Anomaloglossus meansi* sp. n. Abbreviations are defined in the text. Means ± SD are followed by the range in parentheses.

Character	Male (n = 3)	Female (n = 5)	Juvenile (n = 3)
SVL	18.53±0.35 (18.15–18.86)	19.15±1.48 (17.66–21.26)	12.72±2.13 (10.69–14.94)
HW	5.89±0.09 (2.19–2.73)	6.11±0.24 (5.81–6.31)	4.28±0.60 (1.48–1.78)
IOD	2.39±0.30 (2.19–2.73)	2.41±0.20 (2.17–2.72)	1.61±0.15 (1.48–1.78)
HL	5.56±0.31 (5.32–5.91)	5.48±0.28 (5.13–5.81)	3.79±0.60 (3.13–4.29)
EN	1.63±0.04 (1.60–1.68)	1.68±0.11 (1.48–1.75)	1.17±0.26 (0.96–1.41)
SL	2.79±0.02 (2.77–2.82)	2.81±0.17 (2.56–3.00)	2.05±0.30 (1.76–2.36)
EL	2.37±0.17 (2.17–2.48)	2.36±0.13 (2.22–2.51)	1.83±0.13 (1.73–1.89)
TYM	1.08±0.04 (1.03–1.12)	1.06±0.14 (0.86–1.24)	0.69±0.15 (0.58–0.86)
IND	2.44±0.05 (2.39–2.48)	2.60±0.16 (2.37–2.82)	1.83±0.24 (1.59–2.07)
TSL	1.24±0.10 (1.15–1.35)	1.41±0.10 (1.29–1.53)	0.87±0.17 (0.74–1.06)
HAND I	3.12±0.20 (2.96–3.35)	3.25±0.13 (3.10–3.45)	1.89±0.70 (3.10–2.69)
HAND II	3.13±0.06 (3.07–3.2)	3.30±0.14 (3.07–3.45)	2.34±0.39 (2.06–2.78)
HANDIII	4.68±0.10 (4.26–4.44)	4.84±0.19 (4.17–4.66)	3.04±0.46 (2.76–3.57)
HAND IV	3.10±0.10 (3.00–3.2)	3.14±0.12 (2.93–3.22)	2.15±0.31 (1.84–2.46)
WFD	0.57±0.07 (0.52–0.65)	0.55±0.06 (0.52–0.65)	0.43±0.06 (0.37–0.47)
FAL	4.27±0.40 (3.81–4.56	4.41±0.41 (3.97–5.04)	2.54±0.84 (1.94–3.51)
THL	8.29±0.42 (7.80–8.54)	8.85±0.28 (8.52–9.18)	5.37±1.07 (4.61–6.60)
TIL	8.45±0.33 (8.07–8.66)	9.09±0.35 (8.61–9.48)	6.13±1.13 (5.12–7.35)
TAL	4.47±0.21 (4.26–4.68)	4.56±0.38 (4.05–5.02)	3.04±0.73 (2.50–3.86)
FL	7.2±0.38 (7.07–7.45)	7.86±0.38 (7.53–8.48)	4.89±1.27 (3.82–6.29)
WTD	0.72±0.06 (0.67–0.78)	0.68±0.06 (0.61–0.76)	0.43±0.03 (0.42–0.46)

There is substantial variation in colour among preserved individuals, obviously due to preservation artefact (CPI11000 for instance is much lighter than all other individuals). Lower lip pigmented in all male and juvenile individuals, but only in three out of five females. The interorbital region is usually darker than the dorsal ground colour. A short middorsal dark brown/black longitudinal line usually present in the scapular region. One female (ROM 43329) has a diffuse diamond shape marking on the anterior dorsum. Upper surface of arms and legs is cream to dark brown, with darker markings on legs. Palms and soles are light to dark brown. Flanks vary from cream to very dark purplish brown.

#### Distribution and natural history.

**Figure 5.**
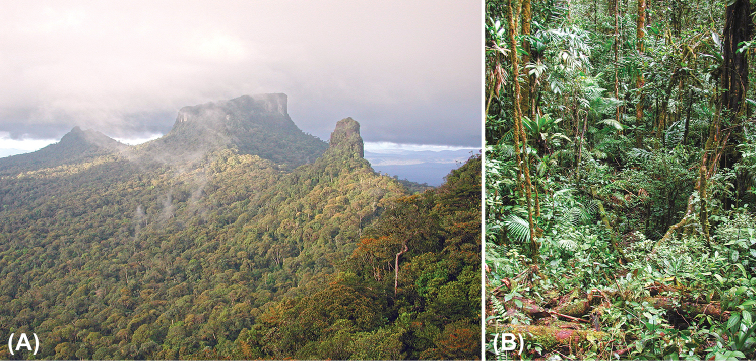
Habitat of *Anomaloglossus meansi* sp. n. on the Wokomung Massif **A** photograph (looking NE) of the highest part of the massif; the plateau in the centre of the photo is the tallest part of the entire Wokomung Massif **B** cloud forest at about 1385 m elevation, habitat of *Anomaloglossus meansi* sp. n. Photographs courtesy D. Bruce Means.

The only localities documented for the new species are depicted in Figure 2. Specimens were collected in cloud forest (Figure 5), on the ground or low vegetation. Most were collected after nightfall, although one adult and one juvenile were collected during daylight. Specimens were collected on mountain flanks, not summits; at 1490 m on Ayanganna, and at 1234 m, 1371 m and 1411 m on Wokomung. The majority of specimens (eight) were collected at 1234 m on Wokomung. Fewer were collected at higher elevations; only one each at 1490 m on Ayanganna, 1371 m and 1411 m on Wokomung. This may have been because of habitat differences; high-canopy open forest at lower elevation and dense, low-canopy vegetation at higher elevations (see Discussion).

#### Etymology.

It is a great pleasure to name this new species after our friend and colleague D. Bruce Means, indefatigable explorer of the “islands in the sky”, and who collected one specimen of the new species and contributed with photographs and data. Thanks to his extensive fieldwork, Bruce Means greatly contributed to our understanding of the distribution, ecology, and taxonomy of Pantepui amphibians and reptiles. The specific epithet should be treated as a noun in the genitive case.

#### Phylogenetic relationships.

The new species was recovered sister to *Anomaloglossus praderioi* by Grant et al. (2006, 2017) and Kok et al. (2012) (see Figure 1). Uncorrected *p* distance in the “barcoding” fragment of the 16S rRNA gene [Vences et al. (2005); based on the sequences used in Grant et al. (2017) and calculated in PAUP 4.0a161 (Swofford 2002)] is 4.3-4.8% between *Anomaloglossus praderioi* and *A. meansi* sp. n. Genetic divergence between populations of *A. praderioi* from the slopes of Roraima-tepui and Maringma-tepui is 0.2%, whereas divergence between populations of *A. meansi* sp. n. from Mount Ayanganna and the Wokomung Massif is 0.9–1.0%.

#### Conservation status.

*Anomaloglossus meansi* sp. n. is only known from four localities and the 11 specimens used in the description. Virtually nothing is known about its ecology, breeding behaviour and population density. Given the uncertainty on its population status we suggest *Anomaloglossus meansi* sp. n. to be listed as Data Deficient according to the IUCN Red List category guidelines (2014).

#### Discussion.

Although Ayanganna and Wokomung are close neighbours, the habitats on their slopes are not exactly similar. The slopes of Ayanganna are a series of relatively flat poorly drained plateaus alternating with steeper slopes. Collecting activities were concentrated on the plateaus, where the vegetation consists of dense, low-canopy high-tepui forest, with a dense understory of woody shrubs and large terrestrial bromeliads (MacCulloch and Lathrop 2009).

The slopes of Wokomung have no large flat plateaus. Habitat at the collecting sites consists of well-drained slopes covered in lower montane cloud forest with some epiphytes and medium density understory, including scattered terrestrial bromeliads. Streams were common on the slopes (MacCulloch et al. 2006). The majority of specimens were found in this habitat, and this may indicate that *Anomaloglossus meansi* sp. n. prefers this to other habitat types; or is a reflection of collecting effort in these habitats.

Species in the *Anomaloglossus beebei* group are currently only known from east of the Rio Caroní, in the Eastern Pantepui District. *Anomaloglossus rufulus* is restricted to the highlands of the eastern part of the Chimantá Massif in Venezuela, whereas *A. kaiei* has a rather large distribution in the uplands of the Pakaraima Mountains of Guyana (Figure 2). The sister species *A. roraima* and *A. beebei* are allopatric, *A. roraima* being restricted to the highlands of the eastern tip of the Eastern Tepui Chain, whereas *A. beebei* is reported further to the east in the uplands of Kaieteur National Park, the Wokomung Massif and Mount Ayanganna (Figure 2). A similar spatial distribution is detected in the sister species *A. praderioi* and *A. meansi* sp. n., which are also allopatric, with *A. praderioi* reported from the uplands of the Eastern Tepui Chain, whereas *A. meansi* sp. n. is only known further to the east in the uplands of Mount Wokomung and Mount Ayanganna.

As mentioned above, virtually nothing is known about the ecology of *A. meansi* sp. n. Based on its phylogenetic position it is likely this species has an exotrophic, lentic tadpole (Figure 1). Comprehensive ecological data are crucial for the assessment of species conservation status, but these assessments are known for a few species only in the Pantepui region and there is a high risk that population declines remain unnoticed in such remote areas.

Two additional phylogenetically distinct species of *Anomaloglossus* remain to be described in the *megacephalus* group (see Grant et al. 2017; the authors, in progress), but several locally restricted *Anomaloglossus* species probably await discovery (Vacher et al. 2017).

## Supplementary Material

XML Treatment for Anomaloglossus meansi

## Appendix 1

**Comparative material examined**

*Anomaloglossus ayarzaguenai*: Venezuela, Estado Bolívar, Cerro Jaua, MHNLS 12949 (holotype), MHNLS 12950–51 (2 paratypes).

*Anomaloglossus beebei*: Guyana, Potaro‐Siparuni District, Kaieteur National Park, IRSNB 13721–26, 13728–53, ULABG 6817 (ex IRSNB 13727).

*Anomaloglossus breweri*: Venezuela, Estado Bolívar, Aprada-tepui, Cueva del Fantasma, MHNLS 17044 (holotype), MHNLS 17045–46 (2 paratypes).

*Anomaloglossus guanayensis*: Venezuela, Estado Bolívar, Serranía de Guanay, MHNLS 10708 (holotype), MHNLS 10712–10714 (3 paratypes), 10716–10717 (2 paratypes), 10724–10725 (2 paratypes).

*Anomaloglossus kaiei*: Guyana, Potaro‐Siparuni District, Kaieteur National Park, IRSNB 1938 (holotype), IRSNB 1939–64 (26 paratypes), IRSNB 14420–57, ROM 42999; Cuyuni‐Mazaruni District, Wayalayeng, IRSNB 14922–24, Maringma-tepui, IRSNB 14925–31, Mount Wokomung, ROM 43321, ROM 43327, ROM 43330, ROM 43333.

*Anomaloglossus megacephalus*: Guyana, Cuyuni‐Mazaruni District, Maringma-tepui, IRSNB 1986 (holotype), Mount Ayanganna, ROM 39637–38 (2 paratypes).

*Anomaloglossus moffetti*: Venezuela, Estado Bolívar, Sarisariñama-tepui, EBRG 4645 (holotype), EBRG 4646–51 (6 paratypes).

*Anomaloglossus murisipanensis*: Venezuela, Estado Bolívar, Murisipán‐tepui, MHNLS 11385 (holotype).

*Anomaloglossus parimae*: Venezuela, Estado Amazonas, Cerro Delgado Chalbaud, ULABG 4221 (holotype), ULABG 4212–20 (9 paratypes), ULABG 4222–26 (5 paratypes).

*Anomaloglossus parkerae*: Venezuela, Estado Bolívar, Sierra de Lema, Salto El Danto, MHNLS 2901, MHNLS 11088–89.

*Anomaloglossus praderioi*: Guyana, Cuyuni‐Mazaruni District, Maringma-tepui, IRSNB 11403–13; Venezuela, Estado Bolívar, Mount Roraima ULABG 4196 (holotype), MHNLS 11272 (paratype), Sierra de Lema, EBRG 5569.

*Anomaloglossus roraima*: Guyana, Cuyuni-Mazaruni District, Wei-Assipu-tepui, IRSNB 15851, IRSNB 15865, IRSNB 15904–11, Maringma-tepui, IRSNB 15864, IRSNB 15883–901; Venezuela, Estado Bolívar, Mount Roraima, ULABG 4197 (holotype).

*Anomaloglossus rufulus*: Venezuela, Estado Bolívar, Amari‐tepui, Chimantá Massif, MHNLS 10361 (holotype).

*Anomaloglossus tamacuarensis*: Venezuela, Estado Amazonas, Sierra Tapirapecó, north base of Pico Tamacuari, MBUCV 6430–33 (4 paratypes).

*Anomaloglossus tepuyensis*: Venezuela, Estado Bolívar, Auyán-tepui, ULABG 2557 (holotype).

*Anomaloglossus triunfo*: Venezuela, Estado Bolívar, Cerro Santa Rosa, Serranía del Supamo, EBRG 4756 (holotype), EBRG 4757–59 (3 paratypes).

*Anomaloglossus verbeeksnyderorum*: Venezuela, Estado Amazonas, Tobogán de la Selva, Municipio Atures, MHNLS 19649 (holotype).

*Anomaloglossus wothuja*: Venezuela, Estado Amazonas, base of Cerro Sipapo, Tobogán del Cuao, EBRG 6689 (holotype).
